# Supplementary material for: United on Sunday: The effects of secular rituals on social bonding and affect
Source: PLoS One. 2021 Jan 27;16(1):e0242546. doi: 10.1371/journal.pone.0242546 (PMC7840012; doi:10.1371/journal.pone.0242546)

United on Sunday: The effects of secular rituals on social bonding and affect

Supplemental Materials

Figure A shows significant correlations between each of social bonding change, positive affect change and the feeling of connection to something bigger, which is to be expected based on previous research. However, negative affect change was not directly correlated with social bonding change but was negatively correlated with positive affect change. Here, we also see that spirituality and religiosity are not directly correlated with bonding change or positive affect change nor, surprisingly, a connection to something bigger.

Supplementary Figure A. Correlation plot showing which items were correlated with which other items I the Sunday Assembly participants. Lines only show for significant correlations (p < .05). Line thickness and opacity relate to magnitude and color to direction of correlation (thicker, more opaque lines show a stronger correlation. Green shows a positive correlation, red a negative correlation).


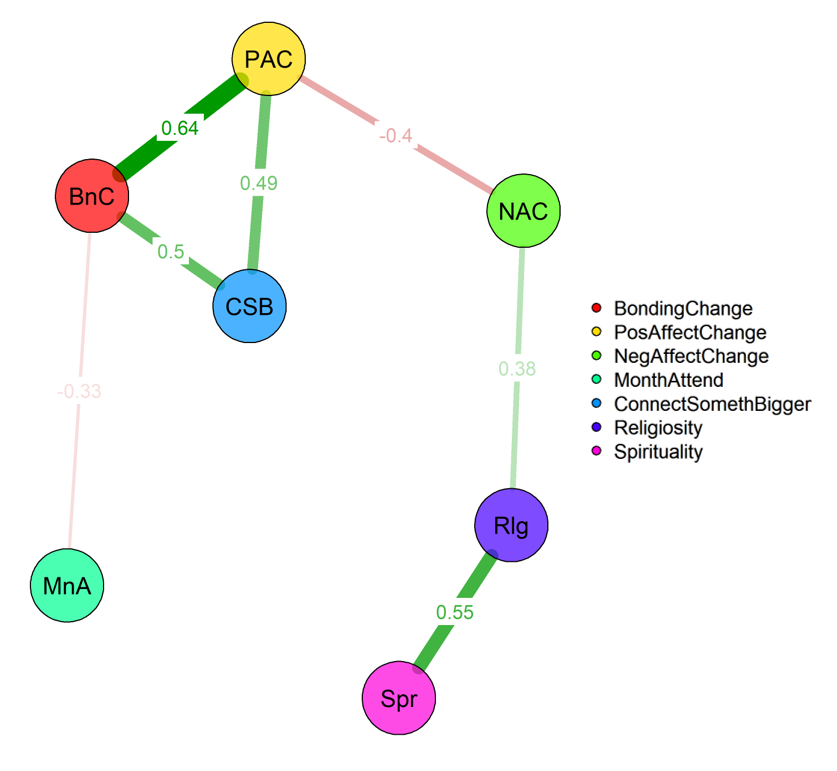


Figure B shows a somewhat different structure compared to the Sunday Assembly data (Figure A), as PANAS- was not correlated to any other variable in the church participants, compared to being correlated with PANAS+ change in Sunday Assembly participants. Moreover, connectedness to God was correlated to PANAS+ change and religiosity in the church participants, compared to connectedness to something bigger being correlated to PANAS+ change and social bonding change in Sunday Assembly participants.

Supplementary Figure B. Correlation plot showing which items were correlated with which other items in the church participants. Lines only show for significant correlations (p < .05). Line thickness and opacity relate to magnitude and color to direction of correlation (thicker, more opaque lines show a stronger correlation. Green shows a positive correlation, red a negative correlation).


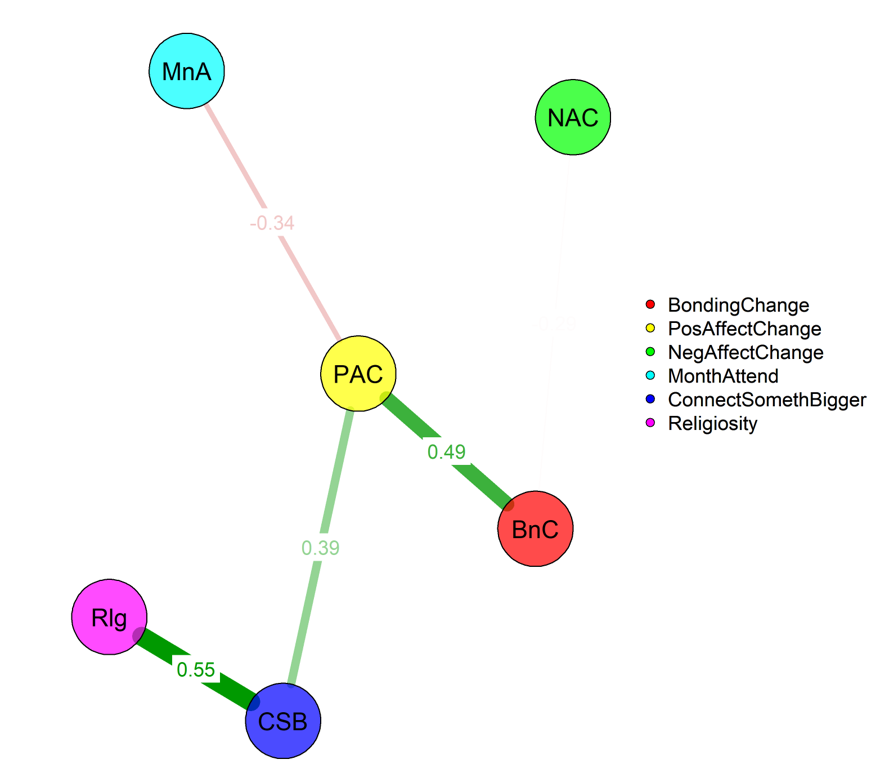

Supplement: S1 Fig — Two supplementary figures showing the correlation plots Sunday Assembly participants (A) and one for church participants (B). (DOCX) [file pone.0242546.s001.docx]
