# Supplementary material for: United on Sunday: The effects of secular rituals on social bonding and affect
Source: PLoS One. 2021 Jan 27;16(1):e0242546. doi: 10.1371/journal.pone.0242546 (PMC7840012; doi:10.1371/journal.pone.0242546)
Supplement: S1 File — (DOCX) [file pone.0242546.s002.docx]

**Communities and the Social Brain survey**

In this survey, please tick or circle the answer that applies. Please tick, cross, or circle only one answer per question, unless it is specifically indicated that multiple answers are possible.

Please feel free to ask the researcher(s) who is/are present if you have any questions at any point.

*For researchers’ use only:*

| Pressure cuff measure before: |  |
| --- | --- |
| Pressure cuff measure after: |  |

**Before the Assembly**

**Question 1**

This scale consists of a number of words that describe feelings and emotions. Please tick for each feeling/emotion the box that represents the extent to which you feel this way ***at the moment***.

|  |  | **Not at all** | **A little** | **Somewhat** | **Moderately** | **Consider-ably** | **Very much** |
| --- | --- | --- | --- | --- | --- | --- | --- |
| 1. | Interested |  |  |  |  |  |  |
| 2. | Distressed |  |  |  |  |  |  |
| 3. | Excited |  |  |  |  |  |  |
| 4. | Upset |  |  |  |  |  |  |
| 5. | Strong |  |  |  |  |  |  |
| 6. | Guilty |  |  |  |  |  |  |
| 7. | Scared |  |  |  |  |  |  |
| 8. | Hostile |  |  |  |  |  |  |
| 9. | Enthusiastic |  |  |  |  |  |  |
| 10. | Proud |  |  |  |  |  |  |

*(The survey continues on the next page, please turn over)*

**Question 1 (continued)**

This scale consists of a number of words that describe feelings and emotions. Please tick for each feeling/emotion the box that represents the extent to which you feel this way ***at the moment***.

|  |  | **Not at all** | **A little** | **Somewhat** | **Moderately** | **Consider-ably** | **Very much** |
| --- | --- | --- | --- | --- | --- | --- | --- |
| 11. | Irritable |  |  |  |  |  |  |
| 12. | Alert |  |  |  |  |  |  |
| 13. | Ashamed |  |  |  |  |  |  |
| 14. | Inspired |  |  |  |  |  |  |
| 15. | Nervous |  |  |  |  |  |  |
| 16. | Determined |  |  |  |  |  |  |
| 17. | Attentive |  |  |  |  |  |  |
| 18. | Jittery |  |  |  |  |  |  |
| 19. | Active |  |  |  |  |  |  |
| 20. | Afraid |  |  |  |  |  |  |

*(The survey continues on the next page, please turn over)*

**Question 2**

At this moment, how connected do you feel to the people in this Sunday Assembly? (Please tick one).

| Not at all (1) | Very slightly (2) | A little (3) | Moderately (4) | Quite a bit (5) | Very much (6) | Extremely (7) |
| --- | --- | --- | --- | --- | --- | --- |
|  |  |  |  |  |  |  |

**Question 3**

Please circle the diagram that best describes your current relationship to this Sunday Assembly, as a whole.


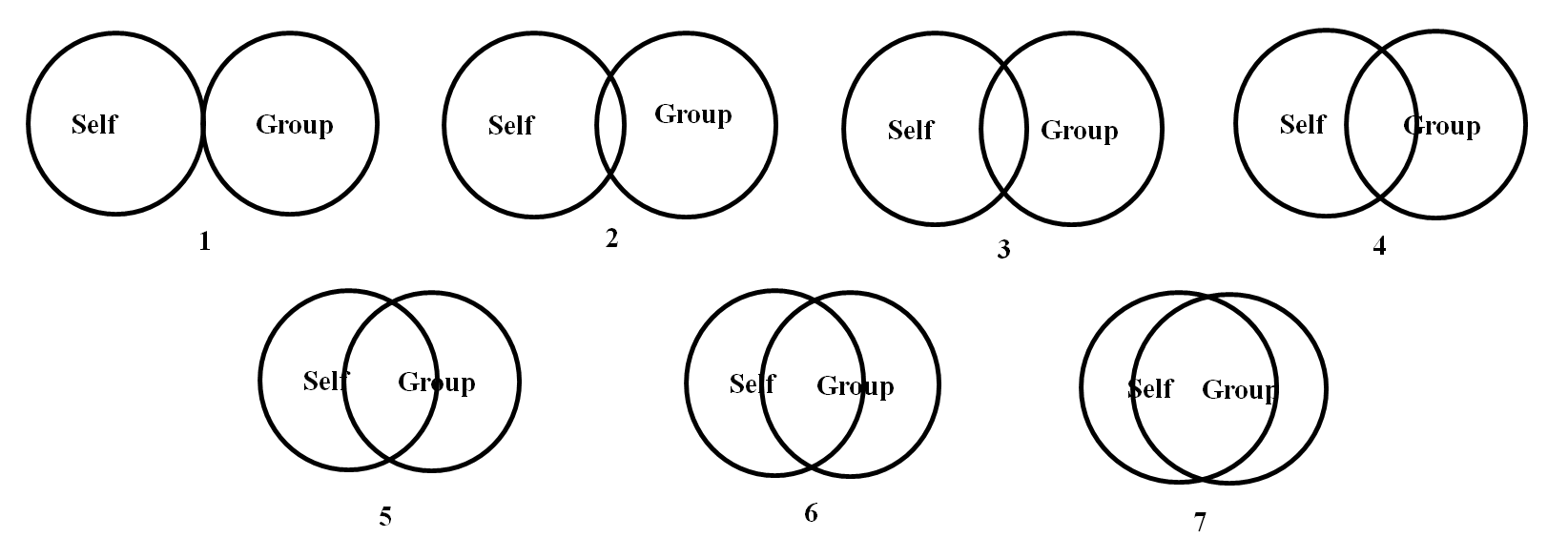


**Question 4**

At this moment, how emotionally close do you feel to the other members of this Sunday Assembly as a whole? (Please tick one).

| Not at all (1) | Very slightly (2) | A little (3) | Moderately (4) | Quite a bit (5) | Very much (6) | Extremely (7) |
| --- | --- | --- | --- | --- | --- | --- |
|  |  |  |  |  |  |  |

*(The survey continues on the next page, please turn over)*

**Question 5**

Thinking about everyone at this Sunday Assembly now, how much do you trust the others in this group? (Please tick one).

| Not at all (1) | Very slightly (2) | A little (3) | Moderately (4) | Quite a bit (5) | Very much (6) | Extremely (7) |
| --- | --- | --- | --- | --- | --- | --- |
|  |  |  |  |  |  |  |

**Question 6**

How much do you like the people at this Sunday Assembly overall? (Please tick one).

| Not at all (1) | Very slightly (2) | A little (3) | Moderately (4) | Quite a bit (5) | Very much (6) | Extremely (7) |
| --- | --- | --- | --- | --- | --- | --- |
|  |  |  |  |  |  |  |

**Question 7**

Thinking about everyone at this Sunday Assembly now, do you feel you have a lot in common with others in this group? (Please tick one).

| Not at all (1) | Very slightly (2) | A little (3) | Moderately (4) | Quite a bit (5) | Very much (6) | Extremely (7) |
| --- | --- | --- | --- | --- | --- | --- |
|  |  |  |  |  |  |  |

**Question 8**

How long have you been going to this Assembly for?

__________years_________months

*(The survey continues on the next page, please turn over)*

**Question 9a**

How religious do you consider yourself to be? Please tick one box.

| Not at all (1) | 2 | 3 | 4 | 5 | 6 | Extremely so (7) |
| --- | --- | --- | --- | --- | --- | --- |
|  |  |  |  |  |  |  |

**Question 9b**

How spiritual do you consider yourself to be? Please tick one box.

| Not at all (1) | 2 | 3 | 4 | 5 | 6 | Extremely so (7) |
| --- | --- | --- | --- | --- | --- | --- |
|  |  |  |  |  |  |  |

**Question 10**

How often do you practice the following?

|  | Never (1) | Rarely (2) | Monthly (3) | Once a week (4) | Several times a week (5) | At least once per day (6) |
| --- | --- | --- | --- | --- | --- | --- |
| a. Engaging in contemplative actions (e.g., prolonged reflection, secular meditation) |  |  |  |  |  |  |
| b. Attending Sunday Assembly or other secular meetings |  |  |  |  |  |  |

Please mark the extent to which the following statement is true or not true for you.

|  | Definitely not true (1) | Tends not to be true (2) | Unsure (3) | Tends to be true (4) | Definitely true of me (5) |
| --- | --- | --- | --- | --- | --- |
| c. I try to carry my secular worldview into all other dealings in life |  |  |  |  |  |

*(The survey continues on the next page, please turn over)*

**Question 11**

Approximately how many people in this Sunday Assembly can you recognise by face?

___________________ person(s)

**Question 12**

Approximately how many people in this Sunday Assembly do you know by name?

___________________ person(s)

**Question 13**

Are there people you know or recognise in this Sunday Assembly that you regularly see at these meetings?

| No (1) |  |
| --- | --- |
| Yes (2) |  |

**Question 14**

Please answer the following question honestly. This information – as all other answers in this survey- will not be linked to your name or identity, but we need this information to ensure our endurance test is appropriate for you as a participant.

|  | **Yes** | **No or N/A** |
| --- | --- | --- |
| Are you currently pregnant? |  |  |
| Do you have arthritis? |  |  |
| Do you have diabetes? |  |  |
| Do you have ADHD? |  |  |

**END OF THE PRE-ASSEMBLY SURVEY**

**Please let one of the researchers know you’re done.**

**END OF THE PRE-ASSEMBLY SURVEY**

**Please let one of the researchers know you’re done.**

**After the Assembly**

**Question 15**

This scale consists of a number of words that describe feelings and emotions. Please tick for each feeling/emotion the box that represents the extent to which you feel this way ***at the moment***.

|  |  | **Not at all** | **A little** | **Somewhat** | **Moderately** | **Consider-ably** | **Very much** |
| --- | --- | --- | --- | --- | --- | --- | --- |
| 1. | Interested |  |  |  |  |  |  |
| 2. | Distressed |  |  |  |  |  |  |
| 3. | Excited |  |  |  |  |  |  |
| 4. | Upset |  |  |  |  |  |  |
| 5. | Strong |  |  |  |  |  |  |
| 6. | Guilty |  |  |  |  |  |  |
| 7. | Scared |  |  |  |  |  |  |
| 8. | Hostile |  |  |  |  |  |  |
| 9. | Enthusiastic |  |  |  |  |  |  |
| 10. | Proud |  |  |  |  |  |  |

**Question 15 (continued)**

This scale consists of a number of words that describe feelings and emotions. Please tick for each feeling/emotion the box that represents the extent to which you feel this way ***at the moment***.

|  |  | **Not at all** | **A little** | **Somewhat** | **Moderately** | **Consider-ably** | **Very much** |
| --- | --- | --- | --- | --- | --- | --- | --- |
| 11. | Irritable |  |  |  |  |  |  |
| 12. | Alert |  |  |  |  |  |  |
| 13. | Ashamed |  |  |  |  |  |  |
| 14. | Inspired |  |  |  |  |  |  |
| 15. | Nervous |  |  |  |  |  |  |
| 16. | Determined |  |  |  |  |  |  |
| 17. | Attentive |  |  |  |  |  |  |
| 18. | Jittery |  |  |  |  |  |  |
| 19. | Active |  |  |  |  |  |  |
| 20. | Afraid |  |  |  |  |  |  |

**Question 16**

During today’s meeting, did you feel connected to something bigger than yourself, like the universe, and/or feel a sense of awe or wonder?

| Not at all (1) | Very slightly (2) | A little (3) | Moderately (4) | Quite a bit (5) | Very much (6) | Extremely (7) |
| --- | --- | --- | --- | --- | --- | --- |
|  |  |  |  |  |  |  |

*(The survey continues on the next page, please turn over)*

**Question 17**

At this moment, how connected do you feel to the people in this Sunday Assembly? (Please tick one).

| Not at all (1) | Very slightly (2) | A little (3) | Moderately (4) | Quite a bit (5) | Very much (6) | Extremely (7) |
| --- | --- | --- | --- | --- | --- | --- |
|  |  |  |  |  |  |  |

**Question 18**

Please circle the diagram that best describes your current relationship to this Sunday Assembly as a whole.


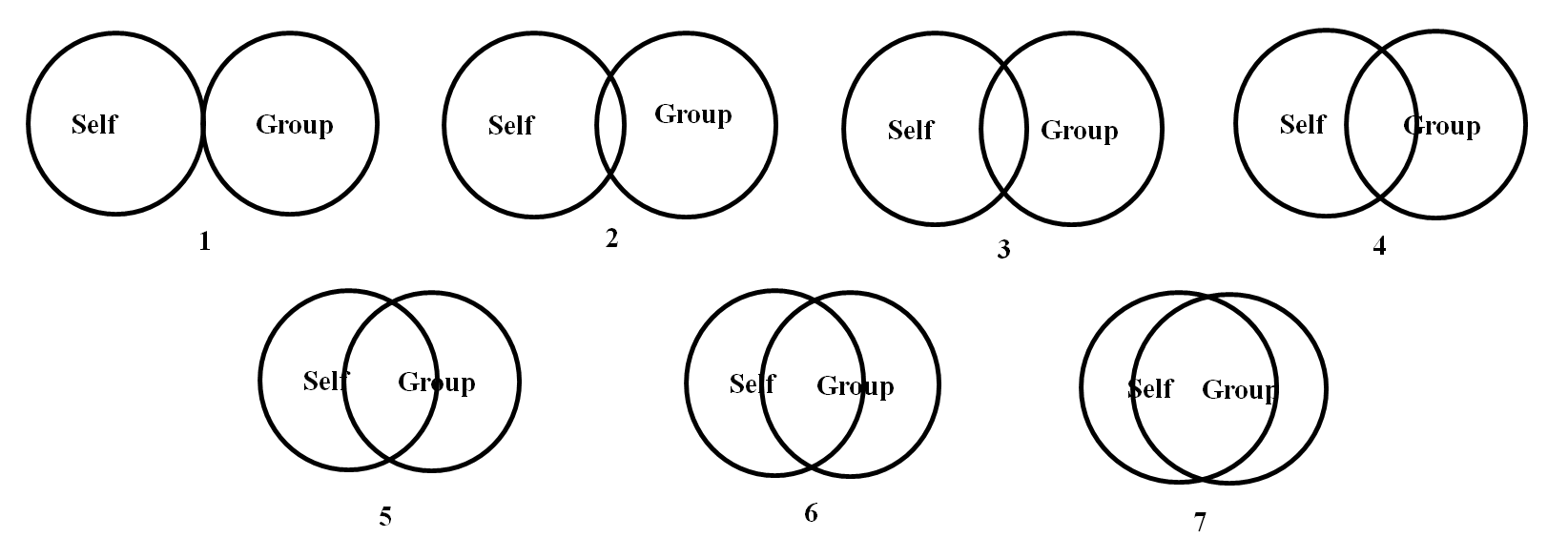


**Question 19**

At this moment, how emotionally close do you feel to the other members of this Sunday Assembly as a whole? (Please tick one).

| Not at all (1) | Very slightly (2) | A little (3) | Moderately (4) | Quite a bit (5) | Very much (6) | Extremely (7) |
| --- | --- | --- | --- | --- | --- | --- |
|  |  |  |  |  |  |  |

*(The survey continues on the next page, please turn over)*

**Question 20**

Thinking about everyone in this Sunday Assembly now, how much do you trust the others in this group? (Please tick one).

| Not at all (1) | Very slightly (2) | A little (3) | Moderately (4) | Quite a bit (5) | Very much (6) | Extremely (7) |
| --- | --- | --- | --- | --- | --- | --- |
|  |  |  |  |  |  |  |

**Question 21**

How much do you like the people in this Sunday Assembly overall? (Please tick one).

| Not at all (1) | Very slightly (2) | A little (3) | Moderately (4) | Quite a bit (5) | Very much (6) | Extremely (7) |
| --- | --- | --- | --- | --- | --- | --- |
|  |  |  |  |  |  |  |

**Question 22**

Thinking about everyone in this Sunday Assembly now, do you feel you have a lot in common with others in this congregation? (Please tick one).

| Not at all (1) | Very slightly (2) | A little (3) | Moderately (4) | Quite a bit (5) | Very much (6) | Extremely (7) |
| --- | --- | --- | --- | --- | --- | --- |
|  |  |  |  |  |  |  |

*(The survey continues on the next page, please turn over)*

**Question 23**

Approximately how many people that you know by name attended the meeting today?

| 0  persons | <5  persons | 5-10  persons | 10-20  persons | 20-30  persons | 40-50  persons | >50  persons |
| --- | --- | --- | --- | --- | --- | --- |
|  |  |  |  |  |  |  |

**Question 24**

Approximately how many people that you know by face attended the meeting today?

| 0  persons | <5  persons | 5-10 persons | 10-20  persons | 20-30  persons | 40-50  persons | >50  persons |
| --- | --- | --- | --- | --- | --- | --- |
|  |  |  |  |  |  |  |

**Question 25**

What is your gender?

| Male (1) |  |
| --- | --- |
| Female (2) |  |
| Non-Binary (3) |  |

**Question 26**

How old are you?

___________________years

*(The survey continues on the next page, please turn over)*

**Question 27**

How much have you exercised in the past 12 hours?

| Not at all (1) | A very slight amount  (2) | A little  (3) | A moderate amount  (4) | Quite a bit (5) | Very much (6) | An extreme amount  (7) |
| --- | --- | --- | --- | --- | --- | --- |
|  |  |  |  |  |  |  |

**Question 28**

How much alcohol have you drunk in past 6 hours?

| Not at all (1) | A very slight amount  (2) | A little  (3) | A moderate amount  (4) | Quite a bit (5) | Very much (6) | An extreme amount  (7) |
| --- | --- | --- | --- | --- | --- | --- |
|  |  |  |  |  |  |  |

**Question 29**

Have you taken any *pain* medication in the past 12 hours? If yes, please specify in as much detail as possible, such as brand/type and quantity/grams.

| **No** | **Yes** – *please specify as much as possible* |
| --- | --- |
|  |  |

**Question 30**

Please rate the importance of the following values as a life-guiding principle for you. Use the 7-point scale in which -1 indicates that the value is opposed to your principles, 0 indicates that the value is not important for you, 2 indicates that the value is important to you, and 5 indicates that the value is of supreme importance for you.

|  |  | Opposed to my principles | Not important | Important | | | Extremely important | Of supreme importance |
| --- | --- | --- | --- | --- | --- | --- | --- | --- |
|  |  | -1 | 0 | 1 | 2 | 3 | 4 | 5 |
| 1 | POWER (social power, authority, wealth) |  |  |  |  |  |  |  |
| 2 | ACHIEVEMENT (success, capability, ambition, influence on people and events) |  |  |  |  |  |  |  |
| 3 | HEDONISM (gratification of desires, enjoyment in life, self-indulgence) |  |  |  |  |  |  |  |
| 4 | STIMULATION (daring, a varied and challenging life, an exciting life) |  |  |  |  |  |  |  |
| 5 | SELF-DIRECTION (creativity, freedom, curiosity, independence, choosing one’s own goals) |  |  |  |  |  |  |  |
| 6 | UNIVERSALISM (broad-mindedness, beauty of nature and arts, social justice, a world at peace, equality, wisdom, unity with nature, environmental protection |  |  |  |  |  |  |  |
| 7 | BENEVOLENCE (helpfulness, honesty, forgiveness, loyalty, responsibility) |  |  |  |  |  |  |  |
| 8 | TRADITION (respect for tradition, humbleness, accepting one’s portion in life, devotion, modesty) |  |  |  |  |  |  |  |
| 9 | CONFORMITY (obedience, honouring parents and elders, self-discipline, politeness) |  |  |  |  |  |  |  |
| 10 | SECURITY (national security, family security, social order, cleanliness, reciprocation of favours) |  |  |  |  |  |  |  |

**Question 31**

How important are the following issues to you personally?

|  |  | Not at all important  (1) | Slightly important  (2) | Moderately important  (3) | Very important  (4) | Extremely important  (5) |
| --- | --- | --- | --- | --- | --- | --- |
| 1 | A close relationship with God |  |  |  |  |  |
| 2 | A strong sense of community |  |  |  |  |  |
| 3 | Animal welfare and animal rights |  |  |  |  |  |
| 4 | Being a good neighbour |  |  |  |  |  |
| 5 | Being welcoming and inclusive |  |  |  |  |  |
| 6 | Care for the environment |  |  |  |  |  |
| 7 | Fair and equal treatment of all people |  |  |  |  |  |
| 8 | Helping the poor |  |  |  |  |  |
| 9 | Honesty |  |  |  |  |  |
| 10 | Interventions in human reproduction |  |  |  |  |  |
| 11 | Sexual morality |  |  |  |  |  |
| 12 | Telling others about your beliefs |  |  |  |  |  |

**Question 32**

These questions ask how you feel about your quality of life and health. Please read each question, assess your feelings, select the best answer for you.

|  |  | Very poor  (1) | Poor  (2) | Neither poor nor good  (3) | Good  (4) | Very good  (5) |
| --- | --- | --- | --- | --- | --- | --- |
| 1 | How would you rate your quality of life? |  |  |  |  |  |
| 2 | How satisfied are you with your health? |  |  |  |  |  |

**Question 33**

The following questions ask about how much you have experienced certain things in the last two weeks.

|  |  | Not at all  (1) | A little  (2) | A moderate amount  (3) | Very much  (4) | An extreme amount  (5) |
| --- | --- | --- | --- | --- | --- | --- |
| 3 | To what extent do you feel that physical pain prevents you from doing what you need to do? |  |  |  |  |  |
| 4 | How much do you need any medical treatment to function in your daily life? |  |  |  |  |  |
| 5 | How much do you enjoy life? |  |  |  |  |  |
| 6 | To what extent do you feel your life to be meaningful? |  |  |  |  |  |
| 7 | How well are you able to concentrate? |  |  |  |  |  |
| 8 | How safe do you feel in your daily life? |  |  |  |  |  |
| 9 | How healthy is your physical environment |  |  |  |  |  |

**Question 34**

The following questions ask about how completely you experience or were able to do certain things I the last two weeks.

|  |  | Not at all  (1) | A little  (2) | Moderately  (3) | Mostly  (4) | Completely  (5) |
| --- | --- | --- | --- | --- | --- | --- |
| 10 | Do you have enough energy for everyday life? |  |  |  |  |  |
| 11 | Are you able to accept your bodily appearance? |  |  |  |  |  |
| 12 | Have you enough money to meet your needs? |  |  |  |  |  |
| 13 | How available to you is the information that you need in your day-to-day life? |  |  |  |  |  |
| 14 | To what extent do you have the opportunity for leisure opportunities? |  |  |  |  |  |

**Question 35**

How well are you able to get around?

| Very poorly  (1) | Poorly  (2) | Neither poor nor well  (3) | Well  (4) | Very well  (5) |
| --- | --- | --- | --- | --- |
|  |  |  |  |  |

**Question 36**

The following questions ask you to say how good or satisfied you have felt about various aspects of your life over the last two weeks.

|  |  | Very dissatisfied  (1) | Dissatisfied  (2) | Neither satisfied nor dissatisfied  (3) | Satisfied  (4) | Very satisfied  (5) |
| --- | --- | --- | --- | --- | --- | --- |
| 1 | How satisfied are you with your sleep? |  |  |  |  |  |
| 2 | How satisfied are you with your ability to perform your daily living activities? |  |  |  |  |  |
| 3 | How satisfied with your capacity for work? |  |  |  |  |  |
| 4 | How satisfied are you with yourself? |  |  |  |  |  |
| 5 | How satisfied are you with your personal relationships? |  |  |  |  |  |
| 6 | How satisfied are you with your sex life? |  |  |  |  |  |
| 7 | How satisfied are you with the support you get from your friends? |  |  |  |  |  |
| 8 | How satisfied are you with the conditions of your living place? |  |  |  |  |  |
| 9 | How satisfied are you with your access to health services? |  |  |  |  |  |
| 10 | How satisfied are you with your mode of transportation? |  |  |  |  |  |

**Question 37**

How similar do you think that your moral values are to the values of others in your Sunday Assembly?

| Very dissimilar  (1) | Somewhat dissimilar  (2) | Neither similar nor dissimilar  (3) | Somewhat similar  (4) | Very similar  (5) |
| --- | --- | --- | --- | --- |
|  |  |  |  |  |

**Question 38**

| No qualifications  (0) | GCSE/O-level or equivalent  (1) | A-Level/high school diploma or equivalent  (2) | Technical/  vocational qualification  (3) | Foundation Degree/Associate’s Degree or equivalent  (4) | Bachelor’s Degree  (5) | Master’s Degree  (6) | Doctorate  (7) |
| --- | --- | --- | --- | --- | --- | --- | --- |
|  |  |  |  |  |  |  |  |

Please indicate from the following the highest educational level you have attained

**END OF THE SURVEY**

**Please let one of the researchers know you’re done.**
